# Phenotypes and subphenotypes of delirium: a review of current categorisations and suggestions for progression

**DOI:** 10.1186/s13054-021-03752-w

**Published:** 2021-09-15

**Authors:** Emily M. L. Bowman, Emma L. Cunningham, Valerie J. Page, Daniel F. McAuley

**Affiliations:** 1 Centre for Public Health, Block B, Institute of Clinical Sciences, Royal Victoria Hospital Site, Queen’s University Belfast, Grosvenor Road, Belfast, BT12 6BA Northern Ireland; 2 Department of Anaesthetics, Watford General Hospital, Vicarage Road, Watford, WD19 4DZ UK; 3 Centre for Experimental Medicine, Wellcome-Wolfson Institute for Experimental Medicine, Queen’s University Belfast, 97 Lisburn Road, Belfast, BT9 7BL Northern Ireland

**Keywords:** Delirium, Inattention, Disorientation, Awareness, Encephalopathy, Pathophysiology, Phenotype, Subphenotype, Endotype, Psychomotor subtype

## Abstract

Delirium is a clinical syndrome occurring in heterogeneous patient populations. It affects 45–87% of critical care patients and is often associated with adverse outcomes including acquired dementia, institutionalisation, and death. Despite an exponential increase in delirium research in recent years, the pathophysiological mechanisms resulting in the clinical presentation of delirium are still hypotheses. Efforts have been made to categorise the delirium spectrum into clinically meaningful subgroups (subphenotypes), using psychomotor subtypes such as hypoactive, hyperactive, and mixed, for example, and also inflammatory and non-inflammatory delirium. Delirium remains, however, a constellation of symptoms resulting from a variety of risk factors and precipitants with currently no successful targeted pharmacological treatment. Identifying specific clinical and biological subphenotypes will greatly improve understanding of the relationship between the clinical symptoms and the putative pathways and thus risk factors, precipitants, natural history, and biological mechanism. This will facilitate risk factor mitigation, identification of potential methods for interventional studies, and informed patient and family counselling. Here, we review evidence to date and propose a framework to identify subphenotypes. Endotype identification may be done by clustering symptoms with their biological mechanism, which will facilitate research of targeted treatments. In order to achieve identification of delirium subphenotypes, the following steps must be taken: (1) robust records of symptoms must be kept at a clinical level. (2) Global collaboration must facilitate large, heterogeneous research cohorts. (3) Patients must be clustered for identification, validation, and mapping of subphenotype stability.

## Background

### Phenotypes

#### Delirium

Lötvall et al. define a phenotype as a set of clinical features in a group of patients who share a common syndrome or condition [1]. Delirium is a clinical syndrome, and therefore a phenotype, characterised by an acute and fluctuating alteration in awareness and cognition resulting from pathophysiological disruption, which may be multifactorial [2–4]. It expresses as a range of symptoms with varying severity and course, affecting patient populations of all ages, but occurring most frequently where patients’ brains are vulnerable and insults severe [3]. A 2020 systematic review and meta-analysis found the occurrence of delirium in the general medical population to be 23%, but incidence varies depending on clinical setting and the diagnostic criteria used [5]. The incidence of delirium in the intensive care unit (ICU) ranges from 45 to 87% [6–8].

Despite an exponential rise in delirium research, understanding of the pathophysiological processes underlying delirium remains low [9]. Regardless of cause, delirium presents as a reasonably common syndrome, particularly when it results from direct or indirect brain injury, but progress is being hindered by a lack of categorical organisation. Robust description which adequately reflects the severity spectrum of the syndrome, outcomes of ranging importance, and the various feasible preventative and management strategies is required [10]. Classifying all delirium presentations under one umbrella term may therefore be hindering proper advancement [11]. There are currently no systematic reviews or articles investigating delirium subphenotypes.

The terminology overlap of delirium and acute encephalopathy has recently been under scrutiny [4, 12]. Acute encephalopathy describes a pathobiological brain process, presenting clinically as subsyndromal delirium, delirium, or coma [12]. The term ‘delirium’ facilitates patient-centred focus allowing screening, preventative measures, and psychological maintenance; however, this clinical phenotype does not account for underlying aetiologies [12]. Conversely, use of ‘acute encephalopathy’ defines cause and pathophysiology, but is ineffective for cases of ambiguous or unknown cause, or for patients whose delirium cause is historical or irreversible [12]. Recent recommendations are against using the terms ‘acute confusional states’, ‘brain dysfunction’, ‘brain failure’, and ‘altered mental status’ [4]. However, any remaining segregation of the literature of delirium and acute encephalopathy could be hindering optimal clinical management [4]. Oldham and Holloway suggest an integrated model where delirium is considered a clinical diagnosis, with encephalopathy used to describe underlying pathways, and the term delirium disorder combining both aspects [12].

#### Subsyndromal delirium

Subsyndromal delirium is also a phenotype, described in 1996 as a condition falling on a continuum between no delirium and DSM-defined delirium [13]. Currently, DSM-5 defines subsyndromal delirium as an attenuated delirium syndrome, which may be challenging to distinguish from mild delirium [14]. It describes symptoms that do not fulfil complete criteria for delirium diagnosis, without specifying which symptoms are present [15–17]. Subsyndromal delirium was investigated in elderly medical inpatients by Cole et al. in 2003 [15] and identified in the critical care setting by Ouimet et al. in 2007 [16]. The current volume of subsyndromal delirium literature is lacking; therefore, a clear conclusion about its association with clinical outcomes in the ICU is impaired. It is currently unknown whether identifying subphenotypes of subsyndromal delirium would be practically or clinically useful [18].

Subsyndromal delirium may be considered as part of a spectrum of delirium severity, when measuring the core diagnostic features [19]. Delirium severity is associated with greater length of hospital stay, 6-month mortality, and higher cost of care [20]. Therefore, these severity ratings are powerful in clinical care, alongside prognosis, and research advancement [21]. A 2019 systematic review found that there are 42 assessment tools for delirium severity and identified 6 of these as high quality, including the Confusion Assessment Method (CAM), Confusional State Examination, Delirium-O-Meter, Delirium Observation Scale, Delirium Rating Scale, and Memorial Delirium Assessment Scale [22]. In critical care, CAM-ICU-7 is often used to measure delirium severity. It measures delirium as not present with a score of 0, subsyndromal 1–2, mild to moderate with a score of 3–5 or severe with 6–7. Of note, individuals experiencing coma receive a score of 7 [23].

#### Aims

We propose building on the existing delirium categorisation framework by identifying potential methods for the integration of the differing delirium phenotypes, and its underlying putative pathways.

**Fig. 1:**
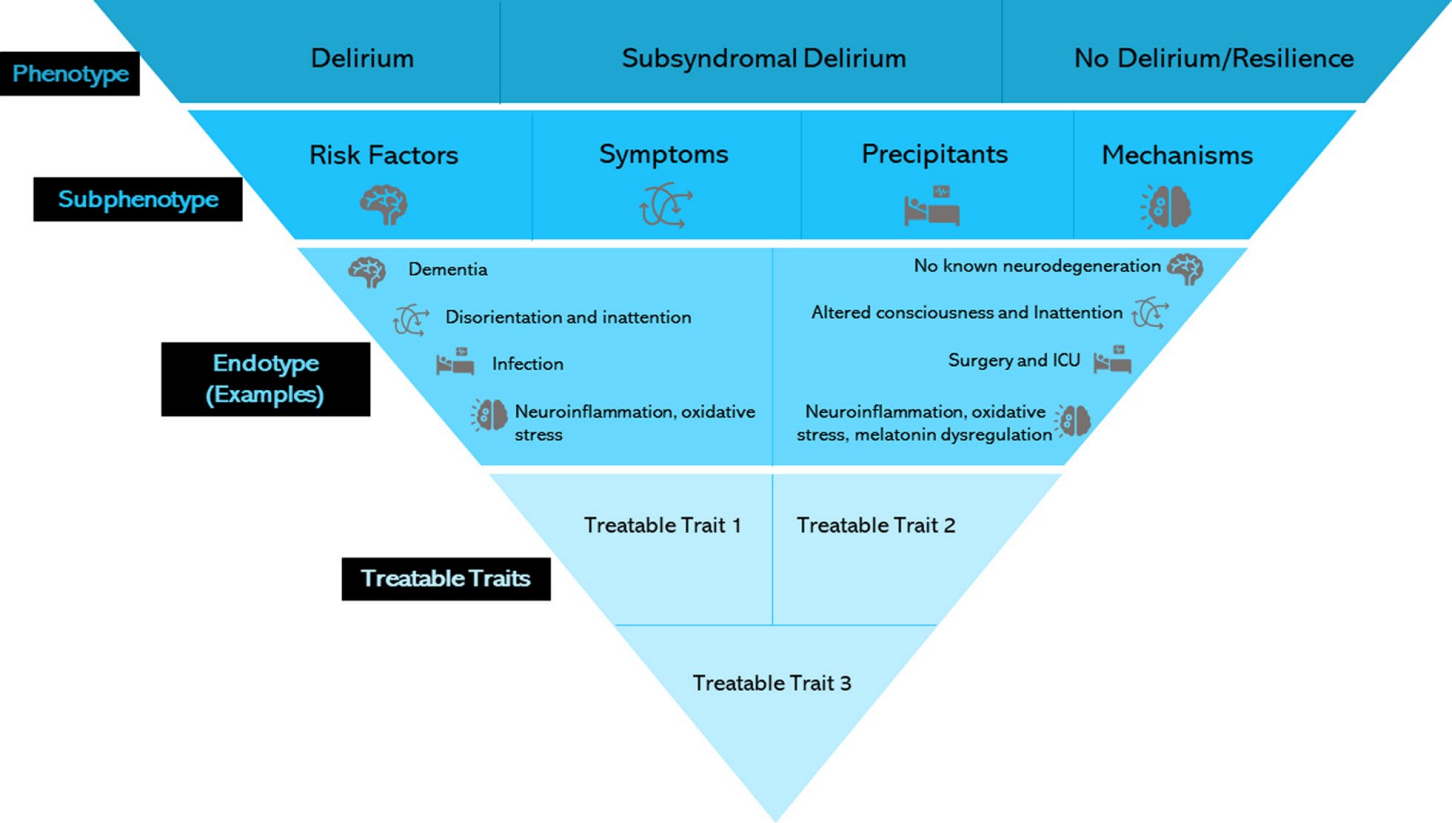
Phenotypes, subphenotypes, endotypes, and treatable traits. Examples of the potential methods for dividing the delirium phenotype into subphenotypes. This may be translated into endotypes, which depend on the characteristics of the subphenotype. Endotype identification may allow the development of treatments targeting specific traits. One person may possess more than one treatable trait

The potential application of subgrouping definitions to delirium is displayed in Fig. 1. This may be done by identifying separate ‘clinical’ subphenotypes, in symptom clusters, and mechanistic subphenotypes, incorporating the pathophysiology of delirium, as shown in Table 1. Integrating these subphenotypes would present an endotype, as shown in Table 1. The recent rise in novel techniques such as genomics, transcriptomics, proteomics, and metabolomics, alongside new tools for data analysis, has allowed increased identification of subgroups of disease, that is subphenotypes [24]. This review aims to highlight the potential alignment of clinical and biological subphenotypes of delirium and suggest how delirium research could benefit from this nomenclature, and to provoke discussion on the subject. We summarise advances in the identification of delirium subphenotypes from various researchers, discussing the implications of these findings on future studies and clinical application, identifying potential barriers in translation to clinical practice, and discussing approaches to overcome these. Pairing the correlation between these subphenotypes may improve knowledge and ability to develop effective delirium treatments. Our framework conveys that delirium is a phenotype, which may be viably categorised into subphenotypes based on one or more clinical or biological traits. Identification of the most suitable subphenotypes may be done by keeping robust and detailed records in many delirium studies, so cluster analysis can be completed and replicated in both homogenous and heterogeneous cohorts. Consequent identification of endotypes, and therefore treatable traits, forms the final layer of this framework.

**Table 1:** A table displaying the definitions of phenotype, subphenotype, endotype, and treatable traits, as described by Lötvall et al. [1]

Term	Definition	Potential application to delirium
Phenotype	A set of clinical features in a group of patients who share a common syndrome or condition.	Altered cognition Inattention Altered awareness Disorientation
Subphenotype	A set of features in a group of patients who share a phenotype. Includes shared risk factors, traits, diagnostic features, expression markers, mortality risk, or treatment response—which distinguishes the group from other patients with the same phenotype.	*Clinical* Shared risk quantification Shared precipitants Specific symptoms, e.g. inattention, agitation Delirium duration Diagnostic features *Defined by pathophysiology* Prominent mechanism Inflammatory/non-inflammatory Melatonin levels Neurotransmitter presence Network connectivity extent Presence of oxidative stress
Endotype	A distinct biological mechanism of disease which is often associated with an anticipated clinical course, shared by a patient subgroup.	Associations between biological putative pathways of delirium and the clinical symptoms which occur as a result
Treatable traits	Subgroup characteristics which may be successfully targeted by an intervention.	Decisions and development of the best course of action for treatment- Treating symptoms Treating the mechanisms which express the symptoms A combination of both

The potential applications of these definitions to delirium are listed, where the phenotype describes the most common clinical domains. Potential subphenotyping methods may be divided by clinical features and by pathophysiological hypotheses, and the endotype is a hybrid between these. Future treatable traits will be decided once the most effective methods are determined. It is important to note that this is a suggested framework for categorisation

### Subphenotypes of delirium

A subphenotype is a set of features in a group of patients who share a phenotype [1]. The subphenotype may include shared risk factors, traits, diagnostic features, expression markers, mortality risk or treatment response, distinguishing the group from other patients with the same phenotype [1]. The potential means of subphenotyping delirium are displayed in Table 1.

#### Clinical subphenotypes

The most common method of subdividing the delirium population is currently by psychomotor subtype. Lipowski first described the hypoactive and hyperactive psychomotor subtypes in 1983 [25], adding the mixed subtype in 1990 [10]. A ‘no subtype’ category exists for patients without psychomotor disturbance [26]. The incidence, severity, and extent of fluctuation observed in the psychomotor subtypes vary; however, the hypoactive and mixed psychomotor subtypes tend to confer worse outcomes and consume more medical resources [3, 27–31]. A recent systematic review of ICU studies reported that the heterogeneity in reporting and methodological quality is limiting robust assessment of outcome differences across subtypes [32].

The delirium traits expressed in the psychomotor subgroups are also specific [28, 33, 34]. In hypoactive delirium, speech is quiet, slow, and less, and the patient may be apathetic, withdrawn, have hypersomnolence, decreased activity, awareness, and alertness, and may experience perceptual disturbances [33, 35]. In hyperactive delirium, the patient may speak louder, faster, and more often. Their actions are faster and less controlled, with increased activity and wandering. There is increased awareness, alertness, restlessness, fear, and higher likelihood of hallucinations and euphoria than in hypoactive delirium. The mixed psychomotor subtype expresses as a transient combination of symptoms from both hyperactive delirium and hypoactive delirium [28]. Further subtyping of psychomotor subtypes has been suggested by severity correlating with outcomes, for example, hypoactive mild, hypoactive severe, mixed, and hyperactive [36]. Hypoactive delirium is seemingly more common than the hyperactive subtype, but the ratio of expressed subtypes differs between studies, with hypoactive delirium occurring in 28–56% hyperactive in 10–47% [35, 37, 38]. Of note, the psychomotor subtype may vary throughout a patients’ hospital stay [37]. In adult survivors of critical illness, longer durations of hypoactive delirium have been associated with a small increase in difficulty completing essential activities of daily life, which was not observed in hyperactive cases [39]. Systematic reviews have explored the incidence of delirium psychomotor subtypes, as well as their risk factors and outcomes [32, 40]. A 2018 systematic review found that 2,080 of 4,550 delirious patients in ICU experienced the hypoactive subtype, making it the most common subtype [40]. However, to our knowledge, there has not been a systematic review conducted exploring the incidence of specific clinical symptoms in various clinical settings, recorded independently from psychomotor subtype. The authors are currently planning a systematic review to fill this research gap.

Steering away from the use of psychomotor subtypes for delirium categorisation would allow identification of more focussed, specific subphenotypes, widening the opportunity for linking precipitants with the syndrome, with the aim of identifying clusters.

Over fifty delirium assessment tools exist, targeting identification of key clinical features by a combination of interview, observation, history, and cognitive tests [41–43]. Delirium duration and rate of disappearance assessment also vary between patients and clinical settings [44]. The curve of delirium disappearance can be established by plotting delirium cases against time, therefore displaying its fluctuation and resolution [44]. A recent study aimed to assess delirium trajectory by integrating severity and duration, describing five distinct delirium ‘trajectories’, or dynamic symptom phenotypes [19]. Patient membership of these phenotypes predicted 30-day mortality [19]. Therefore, subphenotyping by severity and duration of delirium may aid in outcome prediction.

Clinically, classification of delirium by the presence, absence, or severity of symptoms might allow establishment of relationships between precipitants, risk factors, pathophysiology, and their effects. Different symptom domains may result in ranging prognosis; however, this is an area requiring more studies to identify the scale of this problem. A recent systematic review found that higher mortality levels are associated with presence of altered arousal and inattention during delirium episodes, compared to normal arousal or attentional levels in people with or without delirium [45]. Defined delirium symptom domains in the form of subphenotypes may aid in outcome prediction and treatment stratification [45]. Challenge lies in measuring these symptom domains.

#### Precipitant subphenotypes

Classifying delirium by its insults or indicators of acute brain injury has been explored by Girard et al., 2018, where delirium was classified as hypoxic, septic, sedative-associated, metabolic, or unclassified [46]. A large proportion of the unclassified delirium cases in this study experienced worse long-term cognitive outcomes than the other delirium phenotypes, which may represent an unidentified phenotype or an indication of persistent brain injury [46]. Other delirium-related insults include injury, surgery, CNS disorder, nutritional or hydration deficiencies, sleep deprivation, pain, toxin exposure, drug toxicity, anaesthesia, polypharmacy, and severe illness or medical conditions [47]. Often, multiple risk factors exist dependently, or risk factors may be absent or not identified before delirium presentation [46].

Precipitating risk factors are often setting specific. For example, reduced sleep quality is common in the ICU [48], and associated with delirium [49]. A quality improvement process designed to feasibly promote sleep in critical care has been associated with reductions in night-time noise levels, delirium, and coma [50–52]. This process may be conducted stepwise, beginning with disturbance reduction, moving to non-pharmacological methods, and then adding pharmacological aid for those patients whom did not show sleep improvement after the first two stages [50].

#### Risk factor subphenotypes

Predisposing and precipitating risk factors for delirium often coexist. This interrelationship depends on patient vulnerability and the extent of harmfulness of the insult(s) leading to delirium presentation [53]. For example, delirium may express differently in those who are already cognitively impaired, as a lesser insult is needed for delirium precipitation, in comparison with those who are cognitively intact. Table 2 summarises the most common risk factors for delirium in general medicine, and the additional factors to be considered in post-operative and intensive care unit (ICU) settings [10, 43, 54–60]. Effects from pro-cognitive factors, which promote healthy cognition, may also influence expression [12]. Application of predisposing and precipitating risk factors to the description of delirium subphenotypes would significantly increase understanding of how symptoms might be associated with aetiologies and guide research into delirium’s pathophysiological mechanisms. Robust recording and extensive testing of the presence of risk factors are required, alongside consideration for unknown or unidentified risk factors.

**Table 2:** Predisposing and precipitating risk factors in the general medical, post-operative, and ICU settings [10, 43, 54–60]

	General medicine	Additional operative risks	Additional ICU risks
Predisposing risk factors	Older age Low daily activity levels Immobility Sensory impairment Low levels of education Malnutrition Pre-existent cognitive impairment Frailty Comorbidities Alcohol consumption Visual/Hearing impairment	Cerebral disease Chronic diseases: renal, cardiac, hepatic, or pulmonary Alcohol/sedative-hypnotics addiction History of delirium/functional psychosis Depression Vitamin deficiency Seizures or porphyria	Higher illness severity Unexpected hospital admission
Precipitating risk factors	Acute medical illness Fractures Head injury Trauma Surgery Psychological stress Drug use/withdrawal Urinary catheterisation Longer hospital stay	Drug intoxication/anaesthesia Metabolic disturbance Hemodynamic disturbance Respiratory disorders Infection Acute cerebral disorder Alcohol/sedative withdrawal Intraoperative/post-operative: Sleep deprivation Immobilisation Restraints	Mechanical ventilation (and duration of ventilation) Sepsis Opioids Polypharmacy Circadian rhythm disruption Deep sedation Organ failure

Identification of biomarkers in cerebrospinal fluid (CSF) or blood plasma may aid in identification of patients who are most at risk of developing delirium, for example, pre-operatively [61, 62]. Electro-encephalopathy (EEG) is also a useful biomarker for delirium, both in identifying pre-operative vulnerability and active delirium processes, especially in populations which are difficult to assess, like ICU [63].

#### Mechanisms

Classification of delirium based on expression markers or biological data may provide insight into its underlying mechanisms, potentially leading to more ambitious targeted treatments which are not possible with reliance on classification by clinical traits only. At least six neuropathophysiological hypotheses, which remain poorly understood, have been proposed to precipitate delirium [64]. These theories are complementary and accumulative, rather than competitive [64]. They may translate into potential subphenotypes defined by underlying mechanism. Other phenotyping successes, such as that of asthma, have endotyped groups based on the pathophysiological processes leading to the development and progression of disease and treatment responses [65]. This allows further treatment research to target the relevant patient groups [65]. Applying similar methods to delirium, using well-characterised cohorts with appropriate representation of the spectrum of the syndrome, and incorporating bioinformatics, may see phenotyping success, and eventual development of effective treatments [66]. The neuroinflammation hypothesis may allow for categorisation into inflammatory or neuroinflammatory, and non-inflammatory delirium, as the biomarkers may differ depending on presence of inflammation [67, 68]. High levels of C-reactive protein (CRP), a marker of acute inflammatory response, are independently associated with delirium [69]. In ICU patients, higher levels of CRP and procalcitonin are associated with a longer period of delirium or coma [70]. Additional potential biomarkers for neuroinflammation are plasma pro-inflammatory cytokines including tumour necrosis factor (TNF-α), cortisol, S100B-protein, and interleukins (IL-1β, IL-2, IL-6, IL-8, IL-12) [71–77]. Cytokine levels are measured peripherally from the blood, but brain cytokine levels cannot be quantified readily and low accessibility to the central nervous system restricts this research [78]. A 2008 cross-sectional study investigating pro-inflammatory cytokine levels in elderly patients with hip fractures found that plasma IL-6 and IL-8 levels were higher in patients who developed delirium post-operatively [78]. A 2014 study suggested that IL-1β production in the CNS may be an indicator of early event in the pathogenesis cascade of delirium [79]. However, trace IL levels in CSF and the consequent reliability on extrapolation decrease the reliability of these tests [79]. IL-10 is a marker for non-inflamed patients [67]. It has also been hypothesised that repeated endogenous glucocorticoid release in response to stress contributes to delirium development [80].

The neuronal ageing hypothesis may be observed by examination of brain volume using tools such as molecular resonance imaging which may aid in subphenotype identification [81]. This is due to the effects of age in decreasing cerebral blood flow, capillary density, and therefore diminishing brain volume [82, 83]. In ICU patients, long delirium episodes are associated with diminished brain volume at hospital discharge and three months after, indicating neural atrophy [84]. However, without premorbid imaging, it cannot be determined whether this relationship is cause or effect. Volume loss is concentrated in the hippocampus and superior frontal lobe which are important in memory and behaviour control [84]. White matter disruption was also been associated with deteriorating cognition, which may affect attention, working memory, and executive function [85]. Increased levels of the neuronal injury fluid biomarker, neurofilament light (NfL), are also associated with delirium occurrence, in a dose-dependent relationship.

The oxidative stress hypothesis is derived from evidence of increased hippocampal reactive oxygen species (ROS) associated with delirium during in vivo study [86, 87]. Alternatively, hippocampal levels of superoxide dismutase (SOD), glutathione peroxidase (GSH-Px), and catalase (CAT), species involved in the oxidative stress process, are decreased during delirium [88]. Therefore, quantifying SOD, GSH-Px, and CAT concentration may aid in the identification of pathophysiology. Glucose metabolic dysfunction may also contribute to delirium occurrence [89].

The neurotransmitter hypothesis incorporates the serotonergic, dopaminergic, and cholinergic systems [90]. Limitation of tetrahydrobiopterin (BH_4_)—a cofactor in serotonin, and dopamine production, has been associated with both Alzheimer’s disease and delirium after elective cardiac surgery [90, 91]. Elevated anticholinergic activity is also positively correlated with higher delirium severity [92, 93].

The melatonin dysregulation hypothesis suggests that some of the difference in delirium symptoms could be associated with melatonin levels [94]. A prospective study of hospitalised patients in Israel found that patients with hyperactive delirium had lower levels of 6-sulphatoxymelatonin (6-SMT), the chief metabolite of melatonin, compared to hypoactive [94]. Sleep–wake cycle disruption observed in Alzheimer’s disease is attributable to decreased levels of melatonin and disturbed circadian melatonin rhythm [95].

The network dysconnectivity hypothesis suggests that the clinically expressed symptoms of delirium may be attributable to disruption of functional networks in the brain and altered homeostasis of neural oscillation [96]. The default mode network (DMN) is a task-negative intrinsic connectivity network involving brain regions which increase their activity during the absence of a task [97]. Therefore, it is known as a ‘task-negative’ network, which is correlated with a ‘task-positive’ network, resulting in anticorrelation [97]. The DMN incorporates the posterior cingulate cortex, medial temporal lobe, inferior temporal lobe, and inferior parietal lobule [97]. For example, a 2019 study of differences in the resting-state brain network before and during an episode of delirium found a connectivity between the posterior cingulate cortex and negatively correlated pre-frontal cortex during delirium [98]. It also found reduced connectivity between the subcortical regions, indicating that to maintain consciousness, stable cholinergic, and dopaminergic neurotransmission is required [98]. Reduced network anticorrelation may explain the attention deficit observed in delirium [99]. Neuronal network dysconnectivity is thought to be the final buffer in delirium, regardless of aetiology, where there is reduction in normal cognitive function [100]. This dysconnectivity might be a consequence of altered glucose metabolism [101]. The nature of this buffer, or which areas of the DMN are affected, may provide categorisation opportunity, but as the DMN varies with age, sex, and cognitive function, classifying ‘normal’ is challenging [102].

## Translation of subphenotypes into clinical practice

### Challenges in understanding delirium, multimorbidity, and comparison in subphenotyping

Numerous challenges and limitations must be overcome to translate subphenotypes into clinical practice, involving understanding of delirium as a syndrome, study heterogeneity, multimorbidity, research efforts, and the unknowns of subphenotyping. These challenges are detailed in Table 3, alongside suggestions for overcoming said obstacles. An endotype is a distinct biological mechanism of disease which is often associated with an anticipated clinical course, shared by a patient subgroup [1]. Identification of delirium endotypes would integrate the clinical and biomarker-driven subphenotypes to create a hybrid description and open the possibility of targeted interventions of subgroup characteristics, that is, treatable traits [1]. The challenge of translating subphenotypes into endotypes may also be viewed as a strength of the proposed framework for future application, as new ideas, methods, and collaboration are welcomed, to aid in a research area which is still in its infancy. In the setting of precision medicine, the populations available to be studied will be smaller; therefore, global cooperation will enable research questions to be answered in a timely manner.

**Table 3:** A description of the considerations and challenges involved in the subphenotyping process, with a focus on existent studies and understanding

Challenge/limitation	Explanation	Suggestions and examples
Understanding of pathophysiology	Knowledge of the pathophysiological mechanisms of delirium remains largely hypotheses. There is a lack of understanding in how the biomarkers are regulated and interact. However, it is believed that the mechanisms are complementary or co-dependant, so they may be impossible to isolate.	Large studies using unbiased analytical approaches are required to establish causality of delirium models in individual patients. Samples of specific biomarkers from patients who have been subphenotyped should be analysed and their profiles compared Identification of the putative pathways, and the resultant treatable traits, may be achieved by unsupervised clustering analyses of large delirium datasets recording biological variables. Example: *Methods used in the successful endotyping of asthma *[1] Clinically relevant parameters were identified, and a threshold was established to include or omit patients from each endotype. Cluster analysis was then used to identify each endotype. These methods may be applied to delirium by identifying the most suitable parameters for patient categorisation.
Study of relevant patient populations	Studies in specific medical specialities often cannot be generalised across populations due to their environmental heterogeneity, particularly in the ICU. ICU patients are often heavily sedated and difficult to assess, which sometimes leads to small sample sizes in studies, weakening power of results. Due to the high prevalence of delirium in the older population, loss of study follow-up due to attrition is common [118].	Recruitment of representative clinical populations into large, global research studies.
Logistics, global data sharing and research cooperation	Practical and logistical challenges present limitations: Complications, time restraints, and opinions associated with data sharing may also limit the use of globally generated datasets, therefore preventing appropriate research development and the robust findings. In critical care, resources, time, and staffing are occasionally limited, so the completion of regular diagnostic delirium assessments or sample retrieval may not always be possible, especially during the COVID-19 pandemic. Research waste must be overcome.	Technology must be adopted appropriately to allow ease of data sharing and collaboration. Increased levels of training and employment will alleviate staffing issues. To allow direct comparability between studies, future work should use core outcome sets.
Overcoming heterogeneity	The existing delirium literature contains a high level of heterogeneity, therefore identifying the ‘correct’ delirium subphenotypes poses a challenge. The large range of screening techniques could lead to heterogeneity in diagnostic success, and some tests may not distinguish subsyndromal delirium. Mild cases may also be missed during screening, and earlier studies may include conditions other than delirium due to wider definitions which existed [119].	Reproduction of latent class analysis in many large patient cohorts will highlight the extent of the heterogeneity problem. Comparison of results between cohorts of similar characteristics, for example, similar clinical settings, age groups, risk factors, or precipitants, may allow for the identification of subphenotypes suited exclusively to individual groups or, alternatively, show their reproducibility in differing groups. Common techniques must be adopted in appropriate populations for comparability, and consensus reached on a sensitive, robust, diagnostic technique.
Comparison of subphenotypes	It is plausible that independent subphenotypes of delirium cannot be identified, due to its transient nature and risk factor interaction.	Description of subphenotype interactions or creating hybrids between multiple subphenotypes would facilitate increased understanding of the syndrome’s expression in individuals, which is the aim.
Executing analysis correctly	Different results may be generated during cluster analysis, depending on variables included in the analysis and the specific method used [111].	Example: *Successful subphenotyping in acute kidney injury and ARDS* Accumulation of pre-existing vulnerabilities and insult(s) results in acute kidney injury which may be categorised as pre-renal, renal, post-renal, or a combination [112]. Subphenotyping of ARDS has been completed using latent class and cluster analysis, which identified hypoinflammatory, hyperinflammatory, uninflamed, and reactive ARDS [109, 113]. Similar methods of identification must be implemented in delirium for improved and more efficient identification, with methods reported in detail for comparison and replication.
Subphenotype Stability	It is unknown whether subphenotypes and underlying putative pathways are constant throughout. Subphenotype stability across settings and age groups is also unknown. The transient and fluctuating nature of delirium may be problematic in the process of identifying delirium subphenotypes [2]. There is also limited understanding of how short-term phenotypes translate into long-term outcomes. This knowledge limit persists in ARDS despite the progression of subphenotyping in this condition [120].	Symptom fluctuations may be tracked by consistent delirium monitoring using validated assessment methods, at short time intervals, alongside recording individual patient characteristics. Collection of blood and CSF samples consistently at short time intervals and subsequent biomarker analysis will allow an increased understanding of mechanism stability. Stability of the clinical and mechanism-driven subphenotypes should be compared to expand understanding of the interrelationship between delirium symptoms and their biological pathways. Example: *Tracking of subphenotype stability in two ARDS subphenotypes using latent class and latent transition models* [114]. Subphenotype identification may be feasible in the clinical trial context. Methods for stability tracking may be established after the identification of delirium subphenotypes.
Speed of subphenotype assignment	The speed by which subphenotypes of delirium may be identified is crucial in determining their viability. The current method of categorisation by psychomotor subtype allows for quick assessments without leaving the patient bedside.	Fast subphenotype assignment may be achievable for the possible clinical subphenotypes, with robust recording of aetiologies, comorbidities, and response to treatments. Subphenotypes defined by underlying mechanism currently require more time dedicated to assessment of blood and CSF. Development of point of care testing would aid in alleviating this issue.
Multimorbidity	Multimorbidity presents as a problem in delirium, and the range of terms used to report comorbidities in studies increases heterogeneity [54].	Tools such as the Charlson comorbidity index increase ease of comparison between cohorts and may allow a degree of multimorbidity adjustment in analyses [115]. There are research efforts afoot to improved multimorbidity characterisation, so improvements in identification and implementation should be expected in coming years [116, 117].
Treatment response	The possibility exists that the subphenotypes which are identified in patient cohorts are not prognostic for a treatment response.	In this event, the findings will rule out the suggested characterisation methods and allow for development of further novel research plans to improve delirium categorisation.

#### Methods for subphenotype validation

Shared mortality risk or treatment response of delirium clusters may demonstrate correlation between categorisation and outcomes. Non-pharmacological reduction of delirium risk involves several interventions centred around optimising physiology, promoting cognitive engagement and mobilisation [103, 104]. Demonstration that the categorisation of delirium subphenotypes correlates with outcomes may be done by monitoring response to interventions for improvement of orientation. These interventions may include: wearing glasses or hearing aids, mobilisation, pain control, involving relatives and minimising ward movement, could be used, to in a stepwise manner, to organise delirium into subphenotypes [104]. However, evidence of the effectiveness of these non-pharmacological methods in ICU is low and limited to randomised control trials [105]. Setting-specific interventions may also be considered, like sleep promotion in ICU, and avoidance of excessively deep anaesthesia post-operatively [104]. Use of pharmacological treatment in delirium including antipsychotic agents and anticholinesterase inhibitors has been investigated; however, currently none of these treatments are recommended for use [104, 106]. To warrant categorisation by pharmacological treatment response, beneficial effects must first be proven. The extent of variation of outcome or treatment between each of the subphenotype groups remains to be seen. It is possible that some subphenotypes are more highly associated with dementia, institutionalisation, or death. When accounting for all subphenotypes in a population identified by DSM-5 classification, the outcomes will be the same, but may vary between subphenotypes. This may allow targeted therapeutic interventions by analysing ‘phenotype-dependant treatment response’ [107]. The most effective methods of treatment vary between groups, holding potential to provide extensive opportunity for development of clinical protocol. For example, in Acute Respiratory Distress Syndrome (ARDS), differential treatment response has been shown between subphenotypes [108–110].

### Recommendations for future studies

Assignment of subphenotypes of delirium must be a collaborative, global process. Recommendations on achieving this are detailed in Table 4, following guidelines previously suggested by Reddy et al., in the critical care setting [24], and deriving the key efforts required to overcome the challenges discussed in Table 3. In general, identifying aetiology of delirium is difficult and requires large, robust, and highly powered studies, and pre-delirium patients’ brains are often vulnerable.

**Table 4:** Recommendations for research progression in subphenotype identification

Research recommendation	Actions required
Establishment of subphenotype reproducibility and overlap	Use of large prospective studies with heterogeneous patient cohorts to validate subphenotypes and compare similar subphenotypes.
Establishment of subphenotype stability across clinical settings and patient demographics	The prospective studies must exist of heterogeneous patient cohorts across multiple clinical settings. The studies should be repeated to assess subphenotypes which differ by severity and duration, and studies should also be repeated at multiple time points. Subphenotype-related biomarkers should be compared across various settings where appropriate.
Validation of subphenotyping strategies	Sharing of large datasets and algorithms between investigators, ideally by making data open access. Greater levels of cybersecurity required.
Reduction in research competitivity	Emphasis on collaboration and involvement in publications.

## Conclusion

The identification of subphenotypes of delirium is a task which requires prioritising delirium as a syndrome worth vast clinical attention and research. The most viable subphenotyping approaches should be selected and methods for their translation to clinical practice created. Barriers in delirium diagnosis and heterogeneity of populations must be overcome on an international scale to allow precision and use of the most robust clustering techniques. This advancement will enable new levels in understanding of the underlying putative pathways by which the delirium phenotype arises. We recommend eventual progression from subphenotypes to endotypes by creation of a biological–clinical subtype hybrid. This may be completed by identification of underlying mechanism clusters and their course alongside clinical expression. For definition of treatable traits, the mechanistic differences between the subphenotypes must be clarified to allow targeted treatment. Treatments or interventions may then be developed using in vitro and in vivo models and then tested prospectively to assess clinical value.

## Data Availability

Not applicable.

## Abbreviations

ICU: Intensive care unit
CAM: Confusion assessment method
CSF: Cerebrospinal fluid
EEG: Electro-encephalopathy
TNF-α: Tumour necrosis factor
IL: Interleukin
CRP: C-reactive protein
ROS: Reactive oxygen species
SOD: Superoxide dismutase
GSH-Px: Glutathione peroxidase
CAT: Catalase
BH_4_: Tetrahydrobiopterin
6-SMT: 6-Sulphatoxymelatonin
DMN: Default mode network
ARDS: Acute respiratory distress syndrome
